# GDF15/MIC-1: a stress-induced immunosuppressive factor which promotes the aging process

**DOI:** 10.1007/s10522-024-10164-0

**Published:** 2024-12-06

**Authors:** Antero Salminen

**Affiliations:** Department of Neurology, Institute of Clinical Medicine, University of Eastern Finland, P.O. Box 1627, 70211 Kuopio, Finland

**Keywords:** Ageing, Epigenetics, Immunosenescence, Immunosuppression, Integrated stress response

## Abstract

The GDF15 protein, a member of the TGF-β superfamily, is a stress-induced multifunctional protein with many of its functions associated with the regulation of the immune system. GDF15 signaling provides a defence against the excessive inflammation induced by diverse stresses and tissue injuries. Given that the aging process is associated with a low-grade inflammatory state, called inflammaging, it is not surprising that the expression of GDF15 gradually increases with aging. In fact, the GDF15 protein is a core factor secreted by senescent cells, a state called senescence-associated secretory phenotype (SASP). Many age-related stresses, e.g., mitochondrial and endoplasmic reticulum stresses as well as inflammatory, metabolic, and oxidative stresses, induce the expression of GDF15. Although GDF15 signaling is an effective anti-inflammatory modulator, there is robust evidence that it is a pro-aging factor promoting the aging process. GDF15 signaling is not only an anti-inflammatory modulator but it is also a potent immunosuppressive enhancer in chronic inflammatory states. The GDF15 protein can stimulate immune responses either non-specifically via receptors of the TGF-β superfamily or specifically through the GFRAL/HPA/glucocorticoid pathway. GDF15 signaling stimulates the immunosuppressive network activating the functions of MDSCs, Tregs, and M2 macrophages and triggering inhibitory immune checkpoint signaling in senescent cells. Immunosuppressive responses not only suppress chronic inflammatory processes but they evoke many detrimental effects in aged tissues, such as cellular senescence, fibrosis, and tissue atrophy/sarcopenia. It seems that the survival functions of GDF15 go awry in persistent inflammation thus promoting the aging process and age-related diseases.

## Introduction

In their seminal study in 1997, Bootcov et al. (1997) cloned the human macrophage inhibitory cytokine-1 gene (*MIC-1*) which is a member of the TGF-β superfamily. They used subtraction cloning techniques to identify the genes which were induced in response to the activation of human macrophages. They reported that the expression of the *MIC-1* gene was robustly increased in human macrophages after treatments with IL-1β, TNF-α, or IL-2 cytokines. Interestingly, they observed that the exposure of the recombinant MIC-1 protein markedly suppressed the LPS-induced activation of macrophages. Bootcov et al. (1997) also demonstrated that the MIC-1 protein was synthesized as a propeptide which was cleaved and secreted as a cysteine-rich 25 kDa dimeric protein. These results indicated that the MIC-1 protein was an anti-inflammatory cytokine which suppressed macrophage activation. Subsequent studies have revealed that the MIC-1 protein is also expressed in non-immune cells, not only in macrophages and other immune cells (Böttner et al. 1999a; Human Protein Atlas). Several investigators have demonstrated that the MIC-1 protein is an inducible protein although it is persistently expressed in a wide variety of chronic diseases, especially in tumors but also in many age-related diseases (Wischhusen et al. 2020; Kato et al. 2023; Wan and Fu 2024). Wiklund et al. (2010) described the MIC-1 protein as a novel marker of all-cause mortality.

Subsequently, it was observed that MIC-1 was a multifunctional protein and it was re-named as growth differentiation factor 15 (GDF15) (Böttner et al. 1999a). The *GDF15* gene has also been called the non-steroidal anti-inflammatory drug (NSAID)-induced gene-1 (*NAG-1*) (Baek et al. 2001a) and placental transforming growth factor-β (*PTGFβ*) (Lawton et al. 1997). Currently, it is known that the expression level of GDF15 protein is very low in human somatic tissues, except in the placenta, whereas many stresses, especially those related to inflammatory responses, strongly induce the expression of GDF15 in both immune and non-immune cells. Interestingly, the aging process is associated with a significant increase in the expression of GDF15 in human tissues and an escalating level in the circulation (Fuchs et al. 2013; Tanaka et al. 2018; Liu et al. 2021; Conte et al. 2022; Pence 2022). This seems to be connected to an age-related chronic low-grade inflammation process which has been called the inflammaging state (Franceschi et al. 2000). Several proteomic investigations have revealed that the GDF15 protein is a core factor secreted by senescent cells displaying the senescence-associated secretory phenotype (SASP) and moreover, it has been noted that the level of GDF15 in human plasma correlates with multiple aging traits (Basisty et al. 2020; Schafer et al. 2020; Evans et al. 2024). The SASP proteins contain several pro-inflammatory cytokines, chemokines, and colony-stimulating factors which are commonly associated with the chronic inflammation encountered in age-related diseases (Freund et al. 2010). For these reasons, the GDF15 protein was proposed to be an excellent prognostic marker not only for cancers and chronic diseases but also for the biological aging process itself. Currently, it is known that many age-related signaling pathways are able to stimulate the expression of GDF15 although its role in the aging process still needs to be clarified. An increased expression of GDF15 has been closely associated with the microenvironments of immune tolerance, e.g., diverse tumors (Wischhusen et al. 2020), pregnancy (Segerer et al. 2012; Wischhusen et al. 2020), infections and sepsis (Luan et al. 2019; Ahmed et al. 2022), and many age-related diseases, such as cardiovascular disorders as well as chronic kidney and lung diseases (Kato et al. 2023; Wan and Fu 2024; Lasaad and Crambert 2024). Many recent investigations have revealed that the level of circulating GDF15 is a valid biomarker of the severity of viral infections, e.g., in the context of COVID-19 disease (Ahmed et al. 2022; Bu et al. 2024). Clinical studies have revealed that the level of GDF15 upregulation may even identify patients with high risk of severe COVID-19 disease (Bu et al. 2024). Given that the aging process is characterized by an increase in chronic low-grade inflammation and subsequently immunosuppression and immunosenescence (Pawelec et al. 1998; Fulop et al. 2020; Salminen 2020, 2021a), I will examine in detail the role of GDF15 first as an inducer of anti-inflammatory responses and second as an enhancer of an immunosuppressive state.

### GDF15 is a stress-induced regulator of the aging process

Several decades of research have revealed that stress, either physiological or psychological in its origin, affects the aging process. However, it is the intensity level of the stress which modifies the healthspan and lifespan, i.e., a mild stress enhances the hormetic state which has beneficial consequences, whereas excessive stress accelerates the aging process (Gems and Partridge 2008; Rattan 2008; Polsky et al. 2022; Calabrese et al. 2024). There is clear evidence that hormetic stress has many anti-aging effects; this has been demonstrated in many species from *Caenorhabditis elegans* to human beings (Rattan and Demirovic 2009; Sun et al. 2020; Calabrese et al. 2024). Moreover, there are investigations that many chronic inflammatory states, e.g., chronic infections and cancers, can promote a premature aging process in humans (Cupit-Link et al. 2017; Armenian et al. 2019; Poloni et al. 2022). Currently, it is known that acute and chronic responses as well as the magnitude of GDF15 expression can exert divergent responses which indicates that GDF15 is a pleiotropic factor in various physiological stresses (Johann et al. 2021). This implies not only that there is a complex regulation governing its expression but it also allows the protein to evoke either beneficial or detrimental responses in diverse stress situations.

#### Age-related signaling stimulates the expression of GDF15

Cloning studies revealed that the promoter regions of the human *GDF15* gene contain several sequences for the binding of common transcription factors, such as AP-1/2, ATF3/4, CHOP, EGR1, GATA-1, GCN4, GR, HIF-1, NF-κB, NRF1, p53, PU-1, SP1/3, TEAD, WT-1 (Böttner et al. 1999b; Baek et al. 2001b). Moreover, the *GDF15* gene is a TATA box-driven gene. Furthermore, the expression of the *GDF15* gene is regulated not only by signaling pathways targetting certain transcription factors but epigenetic mechanisms also control the transcription of the *GDF15* gene. Several genome-wide evaluations have revealed that DNA methylation regulates the expression level of the *GDF15* gene (Costa et al. 2010; Kadowaki et al. 2012). These studies indicated that DNA hypermethylation significantly reduces the transcription level of the *GDF15* gene, whereas a demethylation of DNA with 5-aza-2′-deoxycytidine robustly increases the expression of GDF15 in different cell lines and tumors (Costa et al. 2010; Kadowaki et al. 2012). In addition to DNA methylation, there is clear evidence that the acetylation and methylation of histones also regulate the expression of the *GDF15* gene (Yoshioka et al. 2008; Lu et al. 2018). For instance, Lu et al. (2018) demonstrated that the enhancer of zeste homolog 2 (EZH2), a histone-lysine methyltransferase in the polycomb repressive complex 2 (PRC2), inhibited the transcription of the *GDF15* gene by modifying the H3K27me3 site in human NSCLC cell lines and that this promoted tumorigenesis in mice. It is known that the aging process is associated with a significant remodelling of the chromatin landscape, both in DNA and histone methylation (Horvath and Raj 2018; Yi and Kim 2020; Duan et al. 2022). Horvath and Raj (2018) proposed the epigenetic clock theory of aging based on the age-related changes in the DNA methylation pattern. This led to the hypothesis that these epigenetic changes would be promising biomarkers for the evaluation of the biological aging process. For instance, it is known that Krebs cycle intermediates regulate DNA and histone methylation and thus mitochondrial dysfunction can affect the aging process (Salminen et al. 2014a). Moreover, the inducible histone demethylase Jumonji D3 (JMJD3) is a potent enhancer of many immune responses, and it can also trigger cellular senescence (Salminen et al. 2014b). It seems plausible that the age-related changes in global DNA methylation and histone patterns could enhance the expression of the *GDF15* gene that occurs with aging.

**Fig. 1:**
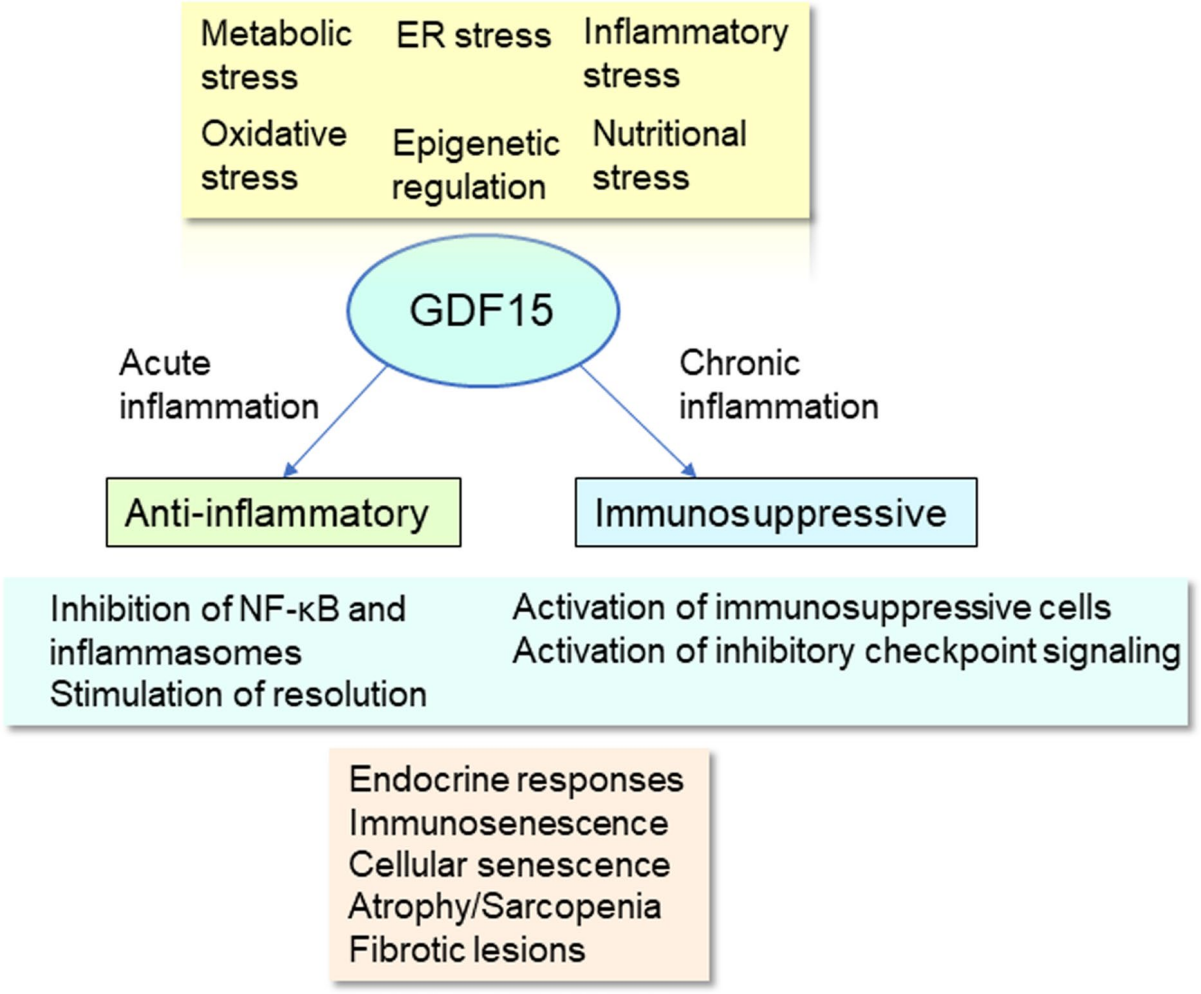
The stimulation of GDF15 signaling promotes different immune responses after acute inflammatory insults and during chronic persistent inflammatory states. Diverse stresses, many of which are age-related, stimulate the expression of GDF15 through different mechanisms which are controlled by epigenetic regulation. In acute inflammation, GDF15 signaling inhibits NF-κB signaling and the functions of inflammasomes, whereas in chronic persistent inflammatory states, GDF15 signaling stimulates the appearance of an immunosuppressive network, a process involving the activation of immunosuppressive cells and inhibitory checkpoint signaling. The activation of GDF15 signaling can generate many pathological responses related to the magnitude and permanency of the inflammatory state. *ER* endoplasmic reticulum, *GDF15* growth differentiation factor 15, *NF-κB* nuclear factor-κB

There is abundant evidence indicating that the integrated stress response (ISR) stimulates the transcription of the *GDF15* gene via the activation of ISR-activated transcription factors, such as the activating transcription factor 4 (ATF4), the C/EBP homologous protein (CHOP), and the X-box-binding protein (XBP1) (Chung et al. 2017a; Zhang et al. 2018; Patel et al. 2019; Fujita et al. 2020; Kim and Lee 2021). In particular, inducers of endoplasmic reticulum (ER) stress and mitochondrial dysfunction are strong enhancers not only of the *GDF15* gene but also of a number of other ISR-related genes (Pakos-Zebrucka et al. 2016) (Fig. 1). Pakos-Zebrucka et al. (2016) have reviewed in detail the inducers and signaling pathways which drive the ISR process and speculated on their possibilities for pharmacological manipulation. Predominantly, the ISR program is a survival mechanism although exposure to excessive or chronic stress can stimulate detrimental effects which pose a threat to the healthy aging process. For instance, the increased expression of GDF15 associated with ISR insults activates signaling via the GDNF family receptor α-like (GFRAL) which mostly is involved in the regulation of energy metabolism (Zhang et al. 2018; Breit et al. 2021). Metabolic stresses, such as chronic high-fat diets or alternatively fasting, are strong inducers of ER stress and thus they elevate the expression of GDF15 (Zhang et al. 2018; Patel et al. 2019) (Fig. 1). Zhang et al. (2018) reported that fasting of mice increased the hepatic expression of GDF15 and moreover, fasting of obese mice exerted beneficial effects by reducing the severity of their hepatosteatosis and insulin resistance. Chung et al. (2017a) demonstrated that the mitochondrial unfolding protein response (UPRmt) in mouse skeletal muscles induced the expression of GDF15 via the activation of CHOP signaling. It seems that the metabolic and pathological outcomes of GDF15 signaling can be either beneficial or detrimental depending on the level of the upregulation and the duration of GDF15 signaling (Johann et al. 2021). There is robust evidence that excessive ER stress and mitochondrial dysfunction can promote the aging process although it is known that a low-grade mitochondrial stress can also have beneficial effects on the aging process (Salminen and Kaarniranta 2010; Barcena et al. 2018; Conte et al. 2019; Burtscher et al. 2023).

The increased expression of GDF15 is a common hallmark of both acute and chronic inflammatory conditions and this is encountered also in many of age-related diseases. It seems that in the presence of inflammatory states, there exist different signaling mechanisms which activate the expression of GDF15. Interestingly, it is known that ER stress and mitochondrial dysfunction, potent inducers of GDF15 (Fig. 1), are also robust inducers of inflammatory responses (Zhang and Kaufman 2008; Salminen et al. 2012; Marchi et al. 2023). The ISR-related inflammatory mediators, such as many of the cytokines and alarmins, can also activate NF-κB signaling which is directly able to transactivate the *GDF15* gene and increase the expression of the GDF15 protein (Ratnam et al. 2017). Moreover, the nuclear factor erythroid 2-related factor 2 (NRF2) and p53 can induce the expression of GDF15, especially in cancer-related inflammation (Yang et al. 2003; Lin et al. 2024). Many phytochemicals have been observed to be potent inducers of the expression of GDF15, e.g., via activation of p53, ATF3, and EGR-1 signaling (Yang et al. 2014). For example, the NF-κB, NRF2, and p53 transcription pathways are known to be involved in the processes driving cellular senescence and tissue aging (Salminen et al. 2008; Rufini et al. 2013; Zinovkin et al. 2022).

Several recent studies have revealed that GDF15 signaling activates AMP-kinase (AMPK) signaling, a master regulator of energy metabolism (Aguilar-Recarte et al. 2023; Jurado-Aguilar et al. 2024; Lu et al. 2024). Aguilar-Recarte et al. (2023) demonstrated that there is a positive feedback loop between the AMPK and GDF15 pathways since the stimulation of AMPK signaling with metformin, an activator of AMPK, increased the expression of GDF15 in mouse liver and skeletal muscle. It seems that an increase in GDF15 signaling is an important drug target of metformin exposure and GDF15 is involved in metformin-induced antidiabetic effects in mice (Aguilar-Recarte et al. 2023). Recently, Jurado-Aguilar et al. (2024) demonstrated that the activation of GDF15 signaling attenuated the TGF-β1/SMAD3 pathway via the activation of AMPK signaling and subsequently it inhibited gluconeogenesis and fibrosis in mouse liver. The GFRAL receptor was not involved in the regulation of GDF15/AMPK signaling. These observations indicate that GDF15 signaling can either activate or inhibit the signaling of TGF-β-driven pathways in a context-dependent manner thus causing sometimes contradictory results. Cooperation between AMPK and GDF15 signaling is an important observation since it is known that AMPK signaling is a potent inhibitor of NF-κB signaling and it has a major role in many inflammatory responses (Salminen et al. 2011a).

There is mounting evidence that GDF15 signaling has a crucial role in the regulation of anti-inflammatory and immunosuppressive processes in inflamed tissues. Luan et al. (2019) characterized GDF15 signaling as a central mediator of host resistance and tissue tolerance in inflammatory conditions. Next, I will shortly describe many GDF-induced non-immune responses related to the aging process and then examine in detail the immune responses induced by GDF15 which have the potential to regulate the aging process.

#### GDF15 signaling promotes aging and age-related diseases

**Fig. 2:**
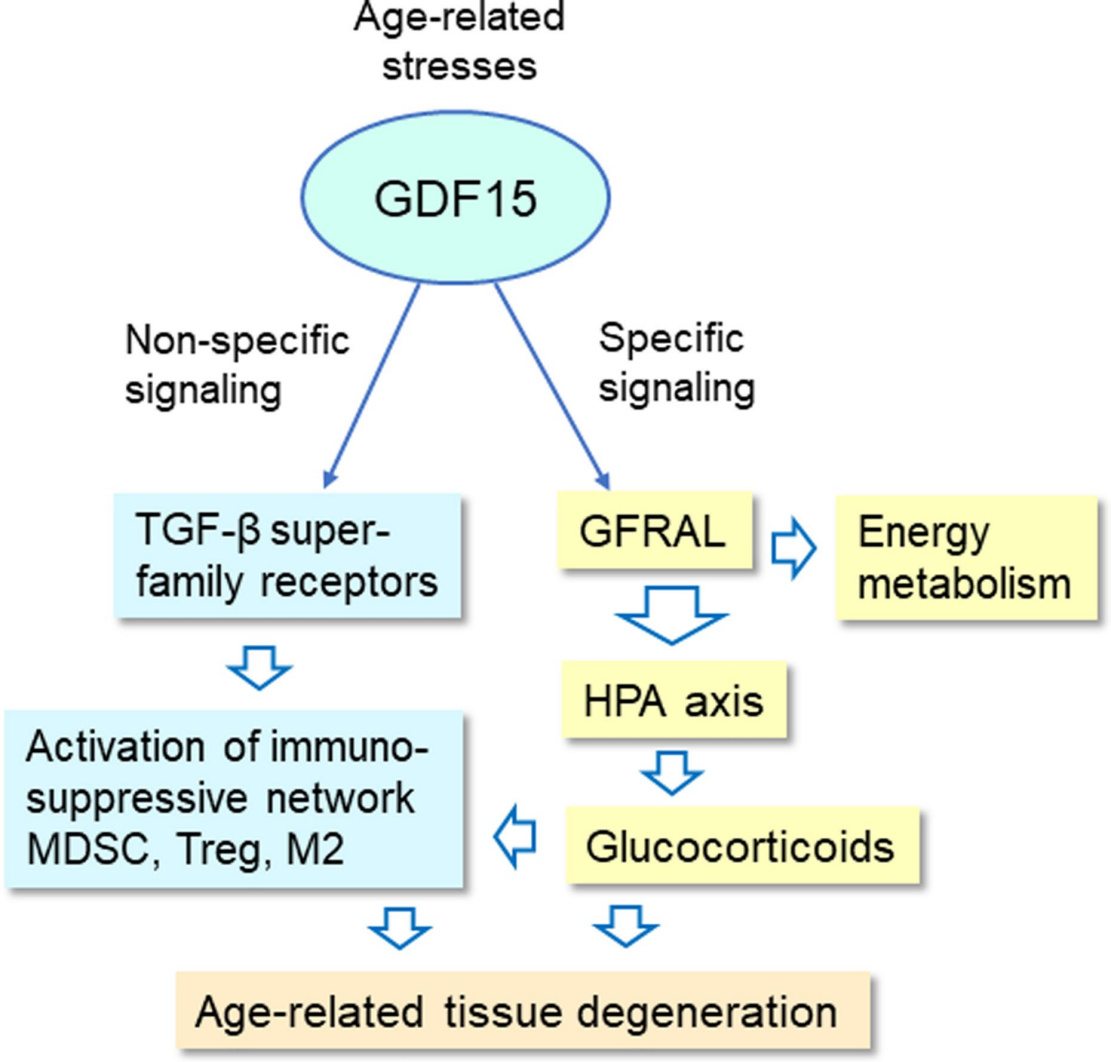
GDF15 signaling regulates many physiological and pathological responses both non-specifically via the receptors of TGF-β superfamily and specifically through the GFRAL/HPA/glucocorticoid pathway. Both the TGF-β-related signaling and glucocorticoids produced via the HPA axis are potent inducers of an immunosuppressive network. GDF15 signaling mediated via the GFRAL receptors also controls energy metabolism via endocrine regulation. The persistent activation of GDF15 signaling, e.g., during the aging process, can induce age-related degenerative changes both via immunosuppression and the release of glucocorticoids. *GDF15* growth differentiation factor 15, *GFRAL* GDNF family receptor alpha like, *HPA* hypothalamic–pituitary–adrenal axis, *M2* M2 macrophage, *MDSC* myeloid-derived suppressor cell, *TGF-β* transforming growth factor-β, *Treg* regulatory T cell

Currently, it seems that the GFRAL receptor is the only specific receptor for the GDF15 protein (Breit et al. 2021) (Fig. 2). The GFRAL receptor is expressed in the neurons of mouse and human hindbrain (Hsu et al. 2017; Breit et al. 2021) although recent studies have revealed that a low-level expression of GFRAL is widely present in restricted cell populations in many murine peripheral tissues (Fichtner et al. 2024). GDF15 affects the energy metabolism of the body by activating the GFRAL receptor, e.g., it enhances fatty acid β-oxidation and reduces appetite thus preventing obesity as well as inducing cachexia and sarcopenia (Hsu et al. 2017; Tsai et al. 2018; Breit et al. 2021). Moreover, there is clear evidence that GDF15/GFRAL signaling activates the hypothalamic–pituitary–adrenal axis (HPA) and thus stimulates the production of glucocorticoids (Cimino et al. 2021) (Fig. 2). In this way, GDF15 is able to control many endocrine functions activated during stress and thus contributes to the development of many metabolic diseases (Iglesias et al. 2023). The role of the HPA axis in the aging process has been under intensive research for decades although it seems to be a complex topic with conflicting results (Gupta and Morley 2014; Gaffey et al. 2016).

It is known that GDF15 signaling is involved in some pathological processes, such as apoptosis, cellular senescence, fibrosis, and atrophy/sarcopenia. These processes are most likely mediated non-specifically via several receptors belonging to the TGF-β superfamily. There is abundant evidence that GDF15 signaling can either induce apoptotic cell death or prevent it in a context-dependent manner. It seems that GDF15 signaling stimulates apoptosis in acute cell damage, e.g., after excessive drug treatments of cancer cells (Wang et al. 2011; Seo et al. 2018; Zhang et al. 2019) or overwhelming ER stress in mouse β-cells (Xu et al. 2022). However, in chronic conditions, GDF15 signaling seems to generate an adaptive response which prevents apoptotic cell death and thus protects tissues, as has been noted with human endothelial cells facing a high glucose challenge (Li et al. 2013). Later, Wang et al. (2022b) demonstrated that NAG-1/GDF15 transgenic mice were resistant to streptozotocin (STZ)-induced type 1 diabetes, i.e., NAG-1/GDF15 signaling inhibited STZ-stimulated apoptosis of β-cells in mouse pancreatic islets and thus attenuated type 1 diabetes. Interestingly, there is clear evidence that resistance to apoptosis increases in many cell populations and tissues with aging, especially in senescent cells, disturbing the maintenance of cellular proteostasis (Salminen et al. 2011b).

Fibrotic lesions in tissues are not only a hallmark of tissue injuries but also of the aging process and the increased presence of tissue fibrosis is a trait of many chronic age-related diseases (Selman and Pardo 2021; Antar et al. 2023). It has been speculated that GDF15 signaling may be involved in the generation of fibrosis since GDF15 is a member of the TGF-β superfamily which includes specific regulators of tissue fibrosis (Ren et al. 2023). The expression of the GDF15 protein is significantly increased in both the circulation and tissues of patients suffering fibrotic diseases, such as idiopathic pulmonary fibrosis (IPF), cardiac fibrosis, and systemic sclerosis (Lambrecht et al. 2014; Rochette et al. 2021; Radwanska et al. 2022). In fact, the serum level of the GDF15 protein has been used as a biomarker of fibrotic diseases. There is robust evidence that GDF15 signaling can evoke many profibrotic properties in different experimental models. For instance, Takenouchi et al. (2020) demonstrated that the expression of GDF15 was abundantly increased in bleomycin-induced pulmonary fibrosis in mice. They also reported that exposure of human lung fibroblasts (WI-38) to the recombinant GDF15 protein stimulated the expression of α-smooth muscle actin (α-SMA) which is an indication on fibroblast activation and generation of fibrosis. Radwanska et al. (2022) revealed that the neutralization of GDF15 significantly attenuated bleomycin-induced pulmonary fibrosis in mice. They also reported that recombinant GDF15 treatment of normal human lung fibroblasts induced the expression of α-SMA via the ALK5 receptors. It seems that GDF15 signaling potentially promotes survival responses in a context-dependent manner although in chronic conditions its responses can become detrimental, such as in fibrotic diseases.

Senescence is a cellular state which has many beneficial functions, e.g., during embryogenesis and tumor suppression, although with aging, the accumulation of senescent cells impairs the function of peripheral tissues (He and Sharpless 2017). Several stresses, especially those associated with the aging process, stimulate the appearance of non-replicating senescent cells within aged tissues. Park et al. (2016) demonstrated that the expression of GDF15 was significantly increased in human aortic endothelial cells (HAEC) made senescent by ionizing radiation. They reported that many biomarkers of cellular senescence could be reversed by the knockdown of GDF15 expression. Park et al. (2016) also revealed that the viral induction of the expression of GDF15 in HAECs stimulated the production of reactive oxygen species (ROS) which triggered a senescence state via signaling through the ERK/p16 pathway. Interestingly, Ha et al. (2019) reported that the GDF15 protein secreted by senescent human endothelial cells improved the function of endothelial colony forming cells (ECFC), the progenitor cells generated from human blood. For instance, GDF15 activated signaling via the AKT, ERK, and SMAD2 pathways and increased the proliferation, migration, and NO production of human ECFCs. It seems that GDF15 signaling might attempt to prevent the vascular dysfunction underpinning many cardiovascular diseases. There are proteomic investigations indicating that the expression levels of GDF15 and several other SASP factors correlate with each other in different senescence models and moreover, the upregulation of SASP biomarkers are associated with advanced chronological age and multiple aging traits (Schafer et al. 2020; Evans et al. 2024).

### GDF15 signaling promotes immune suppressive functions

The GDF15 protein is a pleiotropic factor which can generate opposite responses in different experimental conditions. Moreover, the responses produced by GDF15 signaling can have either beneficial or detrimental outcomes in a context-dependent manner. The immune network is able to display very diverse responses in different experimental environments, e.g., in acute and chronic inflammation as well as combatting both internal and external attacks. Interestingly, there is robust evidence that GDF15 signaling is involved in the function of the immune system and it seems that while GDF15 signaling displays beneficial anti-inflammatory responses against acute insults, in many chronic inflammatory states, its responses seem to be harmful, i.e., it aggravates those diseases.

#### GDF15 is an anti-inflammatory modulator

There is abundant evidence that GDF15 signaling can ameliorate different acute tissue injuries by dampening excessive inflammation such as myocardial ischemia–reperfusion injury (Zhang et al. 2016), sepsis-induced acute lung injury (Lu et al. 2024), and toxin-induced liver injury (Li et al. 2018) (Fig. 1). Experiments conducted with transgenic and knockout GDF15 mice have also confirmed that in acute treatment models, an overexpression of GDF15 reduced the intensity of inflammatory responses and concurrently decreased the severity of tissue injuries, whereas a deficiency of GDF15 augmented inflammatory responses and aggravated tissue injuries (Kim et al. 2013; Abulizi et al. 2017; Chung et al. 2017b; Zhang et al. 2017; Wang et al. 2022c). For instance, Abulizi et al. (2017) demonstrated that treatment of GDF15 knockout mice with lipopolysaccharide (LPS), a sepsis model, significantly increased the inflammatory response and exacerbated injuries in the kidneys and heart as compared to the situation in non-knockout animals. GDF15 deficiency not only increased functional impairments in the kidneys and heart but also augmented the severity of histopathological changes, such as the degree of necrosis and the numbers of infiltrating inflammatory cells. The mortality of the GDF15 knockout mice after LPS treatment was significantly higher than occurred in normal mice. In contrast, Kim et al. (2013) reported that the LPS treatment of the transgenic GDF15 mice evoked a clear decrease in the inflammatory response in comparison to normal mice. Similar anti-inflammatory responses and tissue protection have been observed in other experimental models, e.g., after ischemia reperfusion-induced injuries in the myocardium of transgenic GDF15 mice (Zhang et al. 2017). These studies clearly confirmed the anti-inflammatory properties of GDF15 signaling in acute experiments to both diverse insults and in different tissues. On the other hand, in chronic inflammatory states, such as atherosclerosis, obesity, steatohepatitis, and liver fibrosis, GDF15 signaling can either aggravate inflammation, e.g., in atherosclerosis (Bonaterra et al. 2012; Preusch et al. 2013), or ameliorate inflammation, such as in obesity, steatohepatitis, and liver fibrosis (Kim et al. 2018; Kim and Lee 2021; Li et al. 2023). It seems that in chronic inflammatory conditions, it is not the anti-inflammatory regulation of GDF15 but its other immunomodulatory properties that affect how it will impact on pathogenesis.

In order to confirm that GDF15 signaling inhibited the immune response and not the tissue injury which could reduce inflammatory response, several investigators have performed in vitro studies with immune cells, such as macrophages, and tissue cells to verify the anti-inflammatory properties of GDF15 signaling (Kim et al. 2013; Abulizi et al. 2017; Zhang et al. 2017; Wang et al. 2022b; Lu et al. 2024). It has become evident that the GDF15 protein is a potent anti-inflammatory factor in macrophages (Chung et al. 2017b; Li et al. 2023; Lu et al. 2024), as indicated by its original name, MIC-1 (Bootcov et al. 1997). Moreover, the GDF15-induced anti-inflammatory response, i.e., reductions in the expression and secretion of inflammatory mediators have also been observed in Kupffer cells (Kim et al. 2013), keratinocytes (Zhang et al. 2023), and kidney tubular epithelial cells (Chen et al. 2022). These studies indicated that GDF15 was able to inhibit the expression and secretion of inflammatory mediators both in immune and non-immune cells in an attempt to prevent excessive inflammation in the face of acute insults.

On the other hand, GDF15 can also suppress excessive inflammation by inhibiting the infiltration of leukocytes, such as neutrophils, monocytes, and macrophages, into inflamed tissues. Several investigators have demonstrated that GDF15 was able to inhibit the activation of leukocyte integrins which are required for the endothelial adhesion and subsequent transmigration of these cells into inflamed tissues (Kempf et al. 2011; Artz et al. 2016; Zhang et al. 2016, 2023). It has also been reported that GDF15 can also prevent the activation of platelet integrins and thus inhibit thrombus formation (Rossaint et al. 2013). Kempf et al. (2011) revealed that the GDF15 protein inhibited integrin activation on myeloid cells and reduced their recruitment into infarcted mouse myocardium thus promoting the survival of these animals after an experimentally-induced infarction. Kempf et al. (2011) demonstrated that GDF15 prevented the chemokine-induced clustering of β2 integrins on the surface of polymorphonuclear leukocytes by controlling the interaction between the small GTPases Cdc42 and Rap1. Subsequently, Artz et al. (2016) revealed that the GDF15-induced inhibition of integrin activation on mouse neutrophils was dependent on the formation of ALK5/TGF-βRII heterodimers which inhibited Cdc42/Rap1 signaling. These results indicate that GDF15 exposure can regulate the level of acute inflammation in tissues by inhibiting the recruitment of myeloid cells into inflamed tissues.

The nuclear factor-κB (NF-κB) signaling system is a major regulator of many stress and immune responses not only in acute inflammation but also in chronic inflammatory conditions, including those involved in the aging process and age-related diseases (Hayden et al. 2006; Salminen et al. 2008; Tilstra et al. 2011). Interestingly, given that NF-κB signaling stimulated the expression of GDF15 (Ratnam et al. 2017), several investigators have demonstrated that after different inflammatory insults GDF15 is a potent inhibitor of NF-κB signaling (Ratnam et al. 2017; Li et al. 2018, 2021; Zhang et al. 2023) (Fig. 1). It seems that there exists a negative feedback regulation between the NF-κB and GDF15 signaling pathways. Several studies have revealed that GDF15 signaling inhibited the activation of the TGF-β activated kinase 1 (TAK1), also called MAP3K7, after acute treatments in different experimental models (Ratnam et al. 2017; Li et al. 2018; Zhang et al. 2023). The TAK1 kinase is a canonical protein kinase which activates the IKK/IκB/NF-κB signaling axis and stimulates the nuclear translocation of the p50/p65 transactivation complex. Currently, the molecular mechanisms still need to be unravelled although the role of SMAD2/3 signaling has been proposed to participate in the inhibition of the NF-κB pathway (Ratnam et al. 2017). Recently, Zhang et al. (2024) reported that an elevated GDF15 signaling in human podocytes inhibited the expression of the NEDD4 like E3 ubiquitin protein ligase (NEDD4L) and thus it reduced ubiquitination and the subsequent activation of IKK which is required to trigger NF-κB signaling.

It is known that GDF15 signaling also suppresses the function of inflammasomes which are dependent on the activation of NF-κB signaling (Boaru et al. 2015). Wang et al. (2014) demonstrated that the expression levels of key components of the NLR family pyrin domain containing 3 (NLRP3) inflammasomes, i.e., caspase-1 and the adaptor protein ASC, were significantly lower in the white adipose tissue (WAT) of transgenic GDF15 mice than in their wild-type (WT) littermates. Moreover, the consumption of a high fat diet (HFD) induced a notably lower increase in expression of IL-1β and IL-18, the pro-inflammatory cytokines produced by NLRP3, in the WAT of transgenic mice than in WT counterparts. The transgenic GDF15 mice displayed evidence of a reduced macrophage infiltration into WAT which improved insulin sensitivity. Recently, Wang et al. (2022c) reported that the overexpression of GDF15 in transgenic mice robustly reduced the HFD-induced hepatic activation of the absent in melanoma 2 (AIM2), another type of inflammasome, as compared to WT mice. The increased expression of GDF15 significantly alleviated the HFD-induced obesity and hepatic steatosis in transgenic mice. Recently, it was claimed that an aberrant activation of inflammasomes contributed to the development of many chronic human diseases, such as diabetes and cardiovascular diseases (Yao et al. 2024).

Not only does GDF15 signaling suppress the development of inflammation but it has also crucial functions in the resolution of inflammation (Fig. 1). Macrophages play a major role in the clearance of inflammatory locations from apoptotic neutrophils and in the initiation of tissue repair processes. Macrophage polarization, efferocytosis of apoptotic neutrophils, and an increase in number of immunosuppressive cells are common hallmarks of the resolution of inflammation (Ortega-Gomez et al. 2013). The polarization of pro-inflammatory M1 macrophages into the resolution-phase M2 phenotype has an essential role in the resolution process. There is convincing evidence that GDF15 signaling can promote this polarization of macrophages toward the M2 phenotype in many experimental inflammatory models (Jung et al. 2018; Li et al. 2021; Zhang and Dong 2022; Reyes and Yap 2023). Concurrently, GDF15 signaling inhibits many activities of the pro-inflammatory M1 phenotype, thus enhancing the resolution process. The typical characteristics of alternatively activated M2 macrophages include (i) the redox balance is switched from one producing ROS to an antioxidative state, (ii) the metabolic state is converted from glycolysis to oxidative metabolism, (iii) the phagocytic capacity is improved which promotes the clearance of apoptotic neutrophils, and (iv) the expression of arginase-1, IL-10, and TGF-β is robustly stimulated which enhances resolution and tissue repair. Jung et al. (2018) reported that treatment of human macrophages with recombinant GDF15 increased oxidative metabolism and the M2-like polarization process through the SMAD2 and STAT6 signaling pathways. Li et al. (2021) demonstrated that when human THP-1 and mouse RAW264.7 macrophages were co-exposed to LPS and recombinant GDF15, this combination significantly inhibited M1 polarization of macrophages, while this treatment robustly promoted their M2 polarization. They also reported that GDF15 treatment improved the phagocytic activity and bactericidal functions of macrophages. There is robust evidence that the efficient resolution process requires not only the polarization of macrophages toward the immune suppressive M2 phenotype but it also needs the recruitment and activation of other immunosuppressive cells, such as myeloid-derived suppressor cells (MDSC) and regulatory T cells (Treg) (Ray et al. 2013; Arocena et al. 2014; Proto et al. 2018). It is known that there is an intense crosstalk between immunosuppressive cells as they strive to enhance the resolution of inflammation. For instance, Proto et al. (2018) demonstrated that Tregs promoted the efferocytosis of M2 macrophages during the resolution process. Interestingly, GDF15 signaling can also affect the function of MDSCc and Tregs during the post-resolution period and in chronic inflammatory states. Thus, it does seem that although an increased GDF15 signaling can alleviate acute inflammation and promote its resolution, unfortunately its enhancement of immunosuppression exerts detrimental effects in non-resolving chronic inflammatory conditions.

#### GDF15 is a potent immunosuppressive enhancer

Interestingly, there are many investigations indicating that the immunosuppressive state can continue for weeks after the resolution of acute inflammation (Motwani et al. 2017; Newson et al. 2017). Newson et al. (2017) demonstrated that the post-resolution immunosuppression after a bacterial infection was attributable to the induction of prostaglandin E synthase-1 in macrophages and the generation of prostaglandin E2 (PGE2). PGE2 is a potent immunosuppressive agent, i.e., it suppressed local innate immunity and promoted the differentiation and activity of immunosuppressive MDSCs in infected tissues (Newson et al. 2017). There are tissue-specific differences in the resolution process as well as in those conditions where non-resolving inflammatory states lead to chronic inflammatory diseases (Kanterman et al. 2012; Schett and Neurath 2018). It is known that MDSCs and Tregs are potent immunosuppressive cells which can closely cooperate with M2 macrophages in diverse chronic inflammatory diseases (Gabrilovich and Nagaraj 2009; Amodio et al. 2019; Ou et al. 2023; Goldmann et al. 2024; Huang et al. 2024). There is convincing evidence that the immunosuppressive state in tissues and especially, the occurrence of MDSCs and Tregs can have both beneficial and detrimental effects in chronic inflammatory states (Sanchez-Pino et al. 2021; Gao et al. 2022). In the tumor microenvironment, MDSCs, Tregs, and M2-like tumor-associated macrophages (TAM) are the major immunosuppressive cells which protect tumor cells against immune surveillance (Umansky et al. 2016; Togashi et al. 2019; Basak et al. 2023). Intensive research has also been focused on sepsis-associated immunosuppression and especially on the role of MDSCs and Tregs in the pathogenesis of sepsis (Hotchkiss et al. 2013; Gao et al. 2022; Huang et al. 2024). Conversely, immunosuppression involving the function of MDSCs and Tregs has a crucial protective role in many autoimmune diseases and transplantation tolerance (Shao et al. 2020; Yan et al. 2020; Mikami and Sakaguchi 2023). This is a double-edged sword since while immunosuppressive cells can promote immunosenescence (Salminen 2021a), a process which alleviates autoimmune diseases and transplantation pathology, unfortunately tumor cells and many infections can utilize this immune deficiency to evade immune surveillance and their subsequent clearance from the body.

The immunosuppressive properties of GDF15 have been studied most frequently in cancers, infections, and transplantation as well as somewhat surprisingly in pregnancy (Fig. 1). GDF15 signaling is an important player in the maintenance of an immunosuppressive microenvironment in tumors, thus facilitating tumor growth and metastasis (Ratnam et al. 2017; Rochette et al. 2020; Wischhusen et al. 2020; Muniyan et al. 2022). The GDF15 protein is highly expressed in tumor cells themselves and in the cells surrounding the tumor. For instance, cancer-associated fibroblasts (CAF) are a rich source of GDF15 and they promote the creation of an immunosuppressive microenvironment in prostate cancer (Bruzzese et al. 2014). The hypoxic microenvironment around tumors robustly stimulated the expression of GDF15 via ER stress signaling (Zheng et al. 2020). In addition to the secretion of soluble GDF15 proteins, tumor cells also released GDF15-containing extracellular vesicles which promoted muscle atrophy/cachexia (Zhang et al. 2022). Moreover, hypoxia stimulated the recruitment and polarization of GDF15-producing TAMs which enhanced the chemoresistance of colorectal cancer cells (Zheng et al. 2021). There is clear evidence that tumor growth is also associated with the recruitment and proliferation of immunosuppressive MDSCs, Tregs, and TAMs. It is known that GDF15 signaling can suppress the surveillance of cancer cells and thus enhance their evasion from the immune clearance by cytotoxic NK and CD8^+^ T cells (Roth et al. 2010; Ratnam et al. 2017). Experiments conducted with different models have revealed that the knockdown of GDF15 significantly inhibited the migration, invasion, and proliferation of cancer cells (Li et al. 2020; Zhou and Chen 2024). It seems that GDF15 is a crucial player in the tumor microenvironment and thus the GDF15 protein is a promising target for drug discovery.

The immunosuppressive cells, such as M2 macrophages, MDSCs, and Tregs, not only have a major role in tumor growth but they also undertake essential functions in transplantation and feto-maternal immunotolerance (Köstlin-Gille and Gille 2020; Shao et al. 2020; Juneja et al. 2022). Several investigators have described the important role of GDF15 in the generation of immunotolerance in pregnancy and transplantation biology (Segerer et al. 2012; Zhang et al. 2017; Klein et al. 2023). In fact, the function of GDF15 has received lots of attention in the regulation of Tregs since these cells have a major role in the maintenance of immune tolerance. There is now abundant evidence that GDF15 signaling can enhance immunosuppression by stimulating the generation and activity of Tregs in different experimental conditions. For instance, GDF15 signaling can promote the generation of Tregs either by (i) increasing the expression of indoleamine 2,3-dioxygenase (IDO) (Segerer et al. 2012) which induces the expression of FoxP3/Treg or by (ii) converting naïve CD4^+^ T cells into induced Tregs (iTreg) (Wang et al. 2021). Wang et al. (2021) revealed that GDF15 exposure enhanced the immunosuppressive activity of human natural Tregs (nTreg). They also reported that adult GDF15 knockout mice were defective in their ability to generate competent Treg cells. Moreover, Wang et al. (2021) demonstrated that GDF15 signaling blocked the ubiquitination of FoxP3 protein via the CD48 receptor and thus inhibited its degradation leading to its accumulation into Treg cells. They also reported that in mice, a neutralization of the GDF15 protein with mAb significantly augmented antitumor immunity. Several other researchers have also confirmed that GDF15 signaling enhances the Treg-induced suppression of conventional T cells (Moon et al. 2020). Zhou et al. (2013) also demonstrated that GDF15 treatment suppressed the maturation of human dendritic cells (DC). While GDF15 signaling decreased the secretion of the IL-12 cytokine from human DCs, the secretion of the immunosuppressive compound, TGF-β, notably increased. Zhou et al. (2013) also revealed that GDF15 treatment suppressed the DC-induced T cell stimulation as well as the activation of cytotoxic T lymphocytes. These examples clearly indicate that GDF15 controls the immune suppressive properties not only of T and DC cells but also of macrophages, as described above. Lodi et al. (2021) have reviewed the GDF15-mediated interactions between immune, nonimmune, and cancer cells in the immunosuppressive tumor microenvironment.

The inhibitory immune checkpoint receptor/ligand pathways provide a crucial mechanism for the maintenance of self-tolerance and the regulation of important functions of the immune system, e.g., in diverse chronic inflammatory states (Ravetch and Lanier 2000; Beenen et al. 2022). The inhibitory checkpoints promote immunosuppressive responses, whereas the stimulatory checkpoints enhance the activation of the immune system. In fact, the binding of ligands, either soluble or membrane-bound, to the inhibitory immune checkpoint receptors present on immune cells, e.g., conventional T cells, NK cells, and macrophages, induces an exhaustion of immune cells or alternatively, triggers their differentiation into immunosuppressive cells, such as Tregs, MDSCs, and M2 macrophages (Francisco et al. 2009; Mizuno et al. 2019; Wei et al. 2021; Baldanzi 2022; Salminen 2024a). The main function of inhibitory checkpoint signaling is to balance immune responses and prevent excessive outcomes. Currently, the most frequently examined inhibitory checkpoint system has been the programmed cell death protein-1 (PD-1)/PD-1 ligand-1 (PD-L1) pathway. For instance, the PD-1/PD-L1 pathway suppresses many functions of T and NK cells, e.g., their proliferation and cytotoxic activity (Buckle and Guillerey 2021; Baldanzi 2022). Interestingly, it is known that GDF15 signaling stimulates the expression of the PD-L1protein which consequently suppresses the functions of immune cells via the PD-1 receptor in different experimental models (Peng et al. 2019; Wang et al. 2023). Peng et al. (2019) demonstrated that the expression of the PD-L1 protein was robustly increased in human glioblastoma cell lines. They reported that GDF15 signaling enhanced the expression of PD-L1 via the Smad2/3 pathway in glioblastoma cells. Recently, Wang et al. (2023) revealed that the recombinant GDF15 protein strongly induced the expression of the PD-L1 protein in human gallbladder cancer cells in a time-dependent manner. They also reported that GDF15 treatment increased the expression of the PD-1 protein in human CD8^+^ T cells. In co-culture experiments, they observed that highly PD-L1-positive gallbladder cancer cells were resistant to human activated cytotoxic CD8^+^ T cells. These experiments indicated that the PD-L1 protein induced by GDF15 exposure allowed cancer cells to evade surveillance and immune elimination by cytotoxic T cells. In summary, it seems that GDF15 treatment can promote immunosuppression both by enhancing the activity of immunosuppressive cells and by increasing the function of the inhibitory immune checkpoint pathways.

The activation of immunosuppressive network promoted by GDF15 signaling has clear beneficial effects in excessive chronic inflammatory states but simultaneously it has many harmful outcomes on the integrity of the immune system and the maintenance of homeostasis in peripheral tissues. For instance, malignant cells have “hijacked” diverse immunosuppressive mechanisms to ensure their evasion of immune survaillance in the tumor microenvironments (Nakamura and Smyth 2020; Tie et al. 2022). Moreover, severe inflammatory conditions, e.g., after traumatic injuries and pathogen-induced sepsis, can lead to a state called the persistent inflammation, immunosuppression, and catabolism syndrome (PICS) (Gentile et al. 2012; Horiguchi et al. 2018). This life-threatening inflammatory syndrome can evoke a systemic immunosuppression which exposes the individual to recurrent infections and tumor growth and it can lead to the development of multiple organ failure. The activation of the immunosuppressive network involves changes in the myelopoiesis of immune cells and their recruitment into inflamed tissues as well as an increased differentiation/polarization of regulatory immune cells, such as MDSCs, Tregs, and M2 macrophages (Li et al. 2006; Flavell et al. 2010; Salminen 2020). There are many common hallmarks linked with the increase in immunosuppression, e.g., (i) an increase in secretion of immunosuppressive cytokines, such as IL-4, IL-10, IL-18, and TGF-β, (ii) a release of immunosuppressive factors, such as adenosine and PGE2, (iii) a generation of reactive oxygen and nitrogen species (ROS/RNS), and (iv) an increased expression of amino acid-catabolizing enzymes, such as arginase 1 (ARG1) and indoleamine 2,3-dioxygenase (IDO), in immunosuppressive cells (Li et al. 2006; Whiteside and Jackson 2013; Salminen 2021a, 2021b). In fact, IL-10 and TGF-β as well as being messengers within the cells of the immunosuppressive network, these cytokines also exert many detrimental effects on host tissue homeostasis. For instance, TGF-β exposure not only suppresses the functions of T, B, and NK cells thus enhancing immunosenescence but it can also induce cellular senescence, promote tissue fibrosis, and increase tissue atrophy and sarcopenia (Tominaga and Suzuki 2019; Salminen 2021b; Ren et al. 2023; Lan et al. 2024). Interestingly, these common age-related pathologies are similar to those produced by GDF15 exposure (Fig. 1) which is not surprising considering that GDF15 is a distant member of the TGF-β superfamily.

### Does GDF15 regulate the aging process via immune suppressive responses?

There is mounting evidence that the aging process is connected with an increase in immunosuppression and immunosenescence. Nearly fifty years ago, it was observed that the aging process was associated with a robust increase in the activity of immunosuppressive cells both in mouse spleen and bone marrow (Goidl et al. 1976; Roder et al. 1978; Singhal et al. 1978). Subsequently, several investigators have demonstrated that the number of immunosuppressive cells, e.g., MDSCs, Tregs, and M2 macrophages, is significantly upregulated with aging in the circulation, immune organs, and peripheral tissues of both humans and rodents. Moreover, it is known that the inflammaging process enhances myelopoiesis in the bone marrow which might explain the elevated presence of immature MDSCs in the blood (Pang et al. 2011; Flores et al. 2017). For instance, it was reported that the numbers of MDSCs were noticeably increased with aging in the circulation of elderly people (Verschoor et al. 2013) and mice (Enioutina et al. 2011). Correspondingly, many investigations have revealed that there is a significant age-related accumulation of MDSCs into mouse bone marrow, spleen, and lymph nodes (Grizzle et al. 2007; Enioutina et al. 2011; Flores et al. 2017) as well as into mouse and human skin (Ruhland et al. 2016). Accordingly, it has been observed that there are augmented levels of Tregs with aging in the human circulation (Trzonkowski et al. 2006; Lages et al. 2008). Many researchers have also noted that there is an abundant age-related expansion of Tregs in mouse spleen, lymph nodes, and skin (Lages et al. 2008; Garg et al. 2014; Ruhland et al. 2016). Interestingly, Ruhland et al. (2016) demonstrated that stromal senescence robustly increased the accumulation of MDSCs and Tregs within mouse skin and exposed skin to tumorigenesis. Garg et al. (2014) reported that the Tregs of old mice displayed an enhanced ability to suppress effector T cells which was probably attributable to an increased expression of the FoxP3 protein. There are several studies indicating that the polarization of macrophages toward the M2 phenotype seems to increase with aging in many tissues. For instance, the presence of the M2 macrophages is upregulated not only in mouse bone marrow, spleen, and lymph nodes but also in lungs and skeletal muscles (Jackaman et al. 2013; Wang et al. 2015). However, the M1 phenotype is predominant in aged liver and heart muscle which might reflect the pathology-induced inflammatory changes which occur during aging in these tissues. I have recently reviewed the age-related changes in the immunosuppressive properties of many regulatory immune cells (Salminen 2020).

The aging process is associated with an accumulation of senescent cells within tissues (Yousefzadeh et al. 2020). Recent studies have revealed that senescent cells evade their immune surveillance and elimination from tissues by increasing the expression of ligands for the inhibitory immune checkpoint receptors present mainly in cytotoxic T and NK cells and macrophages (Onorati et al. 2022; Wang et al. 2022a; Salminen 2024a, 2024b). Currently, it is known that senescent cells express diverse ligands for different inhibitory checkpoint receptors and thus they can prevent their own elimination by cytotoxic immune cells (Salminen 2024b). However, the properties associated with the PD-1/PD-L1 axis have been throughly studied in the accumulation of senescent cells (Onorati et al. 2022; Wang et al. 2022a; Salminen 2024a). The expression of the PD-L1 protein clearly increases with aging in many tissues indicative of an expansion of senescent cells (Onorati et al. 2022; Wang et al. 2022a). Onorati et al. (2022) demonstrated that especially the SASP factors upregulated the expression of the PD-L1 protein via the JAK-STAT3 pathway in human fibroblasts. It is known that several aging-associated signaling mechanisms, such as inflammatory mediators, mTOR-related signaling, and the cGAS-STING pathway, can stimulate the expression of PD-L1 (Salminen 2024a). Moreover, Wang et al. (2022a) reported that an intense expression of the PD-L1 protein in mouse pulmonary fibroblasts was able to allow these cells to evade the immune clearance mediated by PD-1-positive, cytotoxic T cells. It seems that GDF15 has an important immunosuppressive role in the accumulation of senescent cells within aged tissues because GDF15 signaling stimulates the expression of the PD-L1 protein (Wang et al. 2023) and furthermore the GDF15 protein is abundantly secreted from senescent cells (Basisty et al. 2020; Schafer et al. 2020; Evans et al. 2024).

Given that GDF15 is a non-specific inducer of TGF-β signaling, many of its immunosuppressive responses could be mediated via the TGF-β pathways (Fig. 2). Immunosuppressive cytokines, such as TGF-β and IL-10, do seem to be involved in promoting many age-related pathological processes, e.g., immunosenescence (Salminen 2021a; Liu et al. 2023), cellular senescence (Tominaga and Suzuki 2019), fibrosis (Ren et al. 2023), and tissue atrophy/sarcopenia (Lan et al. 2024). However, while the aging process is not directly associated with a persistent increase in the expression and secretion of TGF-β and IL-10, there are several reports of a continual increase in the expression of GDF15 during the aging process (Tanaka et al. 2018; Liu et al. 2021; Conte et al. 2022). As described earlier, GDF15 signaling is also able to promote age-related degenerative processes, probably through the receptors of TGF-β superfamily. It seems that GDF15 signaling is able to stimulate immune suppressive responses by activating the immunosuppressive network but concurrently it elicits pathological processes which promote the aging process.

The activation of the hormonal HPA axis is a common response to diverse stressful insults inducing the release of glucocorticoids which control many fundamental physiological processes, such as energy metabolism and the activity of the immune system (de Guia et al. 2014; Cain and Cidlowski 2017) (Fig. 2). Glucocorticoids, e.g., dexamethasone, are potent immunosuppressive hormones and used in the therapy of many chronic inflammatory diseases (Coutinho and Chapman 2011; Giles et al. 2018). Interestingly, Cimino et al. (2021) demonstrated that both the exogenous and endogenous stimulation of GDF15 signaling activated the HPA axis via the GFRAL receptors. They also confirmed that exposure of mice with the blocking antibody to the GFRAL receptor completely prevented glucocorticoid secretion after the administration of GDF15. This seminal investigation established a molecular link between a stress-induced peripheral production of GDF15 and the hormonal feedback response caused by activation of the HPA axis. Interestingly, it is known that glucocorticoids are effective inducers of the major immunosuppressive cells, such as MDSCs (Zhao et al. 2018), Tregs (Kim et al. 2020; Chen et al. 2023; Taves et al. 2023), and M2 macrophages (Kraaij et al. 2011). These results indicate that GDF15 signaling might be able to induce immunosuppressive responses via a specific activation of the GFRAL/HPA axis. It seems that GDF15 signaling is able to stimulate the immunosuppressive network either through the non-specific pathways via receptors of the TGF-β superfamily or specifically through the GFRAL/HPA/glucocorticoid pathway (Fig. 2). Moreover, it should be recalled that glucocorticoids, either acting dependently or independently of GDF15 signaling, can induce age-related degeneration, such as tissue atrophy/sarcopenia, via pathways which are not dependent on the activation of the immunosuppressive network (Sato et al. 2017; Niculet et al. 2020) (Fig. 2).

## Conclusions

There is convincing evidence that GDF15 signaling is a multifunctional stress-related inducer of immune suppressive responses, i.e., it exerts (i) anti-inflammatory responses after acute insults, (ii) is involved in the resolution of inflammatory loci, and (iii) acts as an enhancer of an immunosuppressive microenvironment in chronic inflammatory states. The GDF15 protein is able to suppress inflammatory responses as it is able to non-specifically activate many receptors of TGF-β superfamily and in addition, it can specifically stimulate the GFRAL/HPA/glucocorticoid pathway. Many TGF-β-related responses and glucocorticoid signaling are potent immune suppressive enhancers, i.e., they are well-known as inhibitors of inflammatory pathways as well as having the ability to stimulate the cells of the immunosuppressive network including MDSCs, Tregs, and M2 macrophages. It is not surprising that tumors can benefit from the properties of GDF15 in their fight against elimination by the immune cells of host tissues. Moreover, it is not surprising that the inflammaging process and especially many age-related diseases are associated with a gradual increase in the expression of GDF15. Interestingly, not only inflammatory cytokines but also many age-related stresses, such as ER and mitochondrial stresses, can stimulate the expression of GDF15 without any concomitant induction of the pro-inflammatory cytokines. It seems a paradigm that an increase in the expression of anti-inflammatory GDF15 promotes the pro-inflammatory aging process. Nonetheless, GDF15 signaling also stimulates the function of immunosuppressive cells which not only suppress immune responses but also secrete cytokines, such as IL-4, IL-10, and TGF-β, which have many detrimental effects within tissues. For instance, an increased immunosuppression with aging, either induced by immunosuppressive cells or inhibitory immune checkpoint signaling, can induce immunosenescence, atrophy/sarcopenia, fibrotic lesions, and cellular senescence. It seems that the increased GDF signaling associated with aging is an important player promoting the aging process and age-related diseases.

## Data Availability

No datasets were generated or analysed during the current study.
